# Associations of Job Strain and Health: Differences Among Nurses and Personal Support Workers in Residential Care Homes During the COVID-19 Pandemic

**DOI:** 10.1177/21650799261440222

**Published:** 2026-05-16

**Authors:** Valentina Antonipillai, Edward Ng, Andrea Baumann, Rochelle Garner, Mary Crea-Arsenio, Dafna Kohen

**Affiliations:** 1 Health Analysis and Modelling Division, Statistics Canada; 2 Global Health, McMaster University

**Keywords:** job strain, working conditions, long-term care, health care workers, mental health

## Abstract

**Background:**

The COVID-19 pandemic revealed stressful working conditions experienced by health care workers in long-term care and seniors’ (LTCS) homes worldwide. However, differences in job strain among regulated and unregulated health care workers and its relationship to their health are understudied. This study examined associations between job strain-related working conditions and health changes, exploring differences between nurses and personal support workers (PSWs).

**Methods:**

This cross-sectional study used the Survey of Health Care Workers’ Experiences During the Pandemic (2021). Job strain-related working conditions included workload increases, working overtime, and beyond-role work. Outcomes were workers’ perceived changes in general health and mental health during the pandemic. Descriptive statistics and multivariable logistic regression models were employed, stratified by occupation, and controlled for covariates.

**Findings:**

Job strain prevalence during the pandemic was high, with more nurses reporting job strain-related working conditions (increased workloads, overtime, or beyond-role work) than PSWs. Associations between job strain working conditions and worsened health varied by occupation. Beyond-role work was associated with worsened mental health for both workers, but only worsened general health for nurses, not PSWs.

**Conclusions:**

Differences among nurses and PSWs demonstrate the importance of understanding variations in roles, occupational experiences and policies regulating health care workers.

**Application to Practice:**

These findings support the need for targeted monitoring of mental and general health among nurses and PSWs in LTCS settings. Occupational health practitioners can identify experiences of job strain among staff and provide counsel for solutions, collaborating with management to ensure the LTCS workforce’s health and safety.

## Introduction

The COVID-19 pandemic revealed several challenges faced by health care workers in long-term care and seniors’ (LTCS) homes, including staffing shortages and high levels of burnout in Canada and worldwide (Clarke, 2021; Hoedl et al., 2022; Organisation for Economic Co-operation and Development [OECD], 2020; van Dijk et al., 2022). High mortality in LTCS homes during the early pandemic placed severe pressure on already strained staff (Akhtar-Danesh et al., 2022; CIHI, 2021; OECD, 2021). Previous research demonstrated nursing home staff providing care to over 10 dying residents experienced burnout symptoms, characterized by emotional exhaustion, depersonalization, and reduced personal accomplishments (Puyat et al., 2019). The high infection and mortality rates among older adults in these homes led to increased emotional and psychological distress, and moral injury among workers as they strived to provide care during the pandemic (Embregts et al., 2020; Reynolds et al., 2022).

Workload intensity among LTCS workers, characterized by overtime work and work overload, can be partially attributed to the severe chronic underfunding and understaffing in the sector (Daly et al., 2011; OECD, 2020; Shipman & Hooten, 2007). Data collected almost two decades ago (2004–2006) revealed about half of Canadian direct care workers reported working short-staffed almost every day (Daly et al., 2011); conditions have since declined. During the first year of the pandemic, 66% of long-term care facilities and 60% of seniors’ homes in Canada reported critical staffing shortages, impacting both quality of resident care and health care workers’ safety (Clarke, 2021). With fewer direct care workers to provide long-term care services for an increasingly aging population, remaining workers are consequentially forced to endure experiences of job strain, working longer hours to meet the higher demands of care needs.

Job strain, characterized by high work demands and low job control (Karasek, 1979), has been reported by LTCS workers in OECD countries for decades (OECD, 2020). International evidence suggests LTCS workers in many high-income countries experienced high levels of job strain due to funding constraints, insufficient staffing, high turnover, and poor working conditions that have persisted over time (OECD, 2020; Zúñiga et al., 2015). Prior to COVID-19, LTCS workers faced substantial job strain-related risk factors, such as severe time pressures and an overload of work (Geiger-Brown et al., 2004; OECD, 2020). They reported higher rates of health issues, such as musculoskeletal injuries and poorer mental health, than hospital-based workers and the general population (Alamgir et al., 2007; Hoben et al., 2017; OECD, 2020). Despite these disparities, limited research has documented the extent of job strain-related working conditions during the pandemic, and few studies have quantitatively examined their association with LTCS workers’ health outcomes.

In Canada, over 85% of direct care workers in LTCS facilities are nurses and personal support workers (PSWs; Statistics Canada, 2021). Nurses are regulated workers who are monitored by provincial or territorial regulatory bodies. They carry out administrative tasks and clinical interventions, including dispensing medication, supervising staff, overseeing adherence to care plans, and assessing resident health (Bethell et al., 2018; Prentice et al., 2017). PSWs are unregulated workers, also referred to as health care aides, nursing assistants, or home support workers, who provide personal care, including transferring and repositioning residents with increasingly complex care needs (Ontario Long-term Care Association, 2025). Research has shown PSWs are among the most at risk of musculoskeletal injuries and disability (Alamgir et al., 2007; Baughman & Smith, 2012; Zagrodney et al., 2023). Given differences in roles and responsibilities among workers, particularly during the pandemic, understanding whether job strain prevalence differs across occupational groups within the sector is essential.

Recent evidence suggests that Canada’s LTCS workers experienced declines in physical and mental health during the pandemic (Carazo et al., 2022; Havaei et al., 2021; Voth et al., 2022). However, there is limited information on the factors that place LTCS workers at higher risk of poorer health outcomes, and disaggregated data on unregulated workers, such as PSWs, remain scarce. Differences in roles, expectations, and regulatory oversight between nurses and PSWs necessitate examining job strain and health changes across these groups.

### Job Strain Model in LTCS Homes

Job strain is a form of psychological stress, encompassing physical or psychological responses to workplace stressors. In this study, the stressor refers to working conditions or events creating demands which tax or exceed an individual’s capability or resources to adapt to them (Lazarus & Folkman, 1984). This study employs Karasek’s (1979) Job Strain Model to examine how work environments influence employee health. Karasek (1979) posits job strain results from interactions between high job demands and low job control. Job demands refer to the psychological pressures at work related to load, including work intensity and time constraints, while job control measures decision latitude, which is the degree of autonomy and discretion afforded to employees on the performance of their tasks, as well as the use and development of their skills. Karasek’s (1979) model assumes these two constructs interact in different ways that determine job strain levels. Workers with increased job demands and low control over those demands experience *high-strain employment*, whereas those with fewer work demands and high control experience *low-strain jobs. Active jobs* have high demands and high control, fostering learning and skill development, while *passive jobs* involve low demands and low job control resulting in skill underutilization. Ultimately, the model theorizes that workers may develop poor physical and mental health when they face high demands combined with low autonomy in meeting those demands.

In this study, the model is employed to examine how working conditions that increase demands and reduce control relate to changes in direct care staff health outcomes in LTCS settings. Direct care staff, including nurses and PSWs, faced strenuous working conditions exacerbated by the pandemic. While prior studies conducted in Sweden, Germany, and the US applied Karasek’s model to LTCS workers, they often treated workers as one homogenous group (Backman et al., 2018, 2023; Wendsche et al., 2016; Woodhead et al., 2016). This study extends existing work by comparing job strain experiences across different occupational groups within Canada’s LTCS sector.

### Study Objectives

Decreasing retention rates and high turnover among nurses and PSWs suggest the working conditions related to job strain and their association with worker well-being require investigation. This study examines the prevalence of job strain-related working conditions among nurses and PSWs in Canada’s LTCS homes and assesses associations between job strain and changes in self-reported mental and general health during the COVID-19 pandemic. Specifically, the job strain-related working conditions investigated are increased workloads, overtime, and engagement in work outside expected roles and responsibilities.

## Methods

### Design and Data Sources

A cross-sectional study design was employed using data from Statistics Canada’s Survey on Health Care Workers’ Experiences During the Pandemic (SHCWEP) to understand associations between changes in health and job strain working conditions in the LTCS sector. The survey collected information from health care workers aged 18 years or older, employed in a healthcare setting since the start of the COVID-19 pandemic (March 2020), living in one of the ten Canadian provinces and completed between September 2, 2021, and November 12, 2021. The SHCWEP was designed as a national survey representative of health care workers across provinces who were employed in the sector at the time of the 2016 Census or were registered in health care education programs between 2015 and 2018. The survey was part of the COVID-19 Federal Health Workforce Information Strategy, and its content was developed in consultation with the Public Health Agency of Canada, Health Canada, and the Canadian Institute for Health Information. Data were collected directly from respondents using an electronic questionnaire or computer-assisted telephone interviewing. All questionnaire content and data collection underwent extensive testing (Statistics Canada, 2022). Stratified random sampling of 22,293 health care workers was conducted using the Census 2016 long-form and the Postsecondary Student Information System. Strata are defined by province and occupation group and the response rate was 55% (12,246 completed surveys; Statistics Canada, 2022). Respondents were included in the study sample if their primary job location was in LTCS homes and they provided direct care to residents, such as nurses (including registered nurses, registered practical nurses, nurse practitioners, among others) and PSWs. Those working in other healthcare sectors, such as acute or outpatient care, were excluded. Physicians and allied health professionals represented less than 5% of LTCS workers and were also excluded from the study sample.

### Exposure and Outcome Variables

Job strain is the primary exposure variable conceptualized using Karasek’s (1979) Job Strain Model and includes two key components: workload intensity and job control. In this study, workload intensity was measured using two items: working overtime and increased workloads. Job control was assessed as performing work outside of one’s expected role, termed beyond-role work. The three job strain indicator variables—working overtime, increased workloads, and beyond-role work—are dichotomous (yes or no). The prevalence of job strain was calculated when workers reported at least one of the three job-strain-related working conditions.

Two outcome measures were included: (1) perceived changes in general health and (2) perceived changes in mental health. Responses on a 5-point scale were dichotomized as either “improved/no change” (“much better,” “somewhat better,” or “no change”) or “worsened” (“much worse” or “somewhat worse”) to examine associations between exposures and outcomes using logistic regressions. Only a small proportion of workers reported improved health status (10%–15%).

### Covariates

Covariates were determined from the survey data collected, some of which were collapsed by the authors to ensure sufficient sample sizes for reliable statistical analysis and reduction in variability among small subgroups, especially when disaggregating by occupation (DiStefano et al., 2021). Those of interest included employment characteristics, such as number of years worked in the sector (0–4 years, 5–14 years, or 15 years and over), and number of locations worked (1 or 2+). Working environment characteristics were also controlled for, including facility ownership status (private, public, or unknown), which was coded according to the primary place of work, defined as the location at which an employee worked the most hours. Additionally, exposure to precarious working conditions was adjusted for, as measured by a work precarity indicator assessing whether workers experienced at least two of the following: reduced hours of work, loss of income, and/or unpaid leave (Antonipillai et al., 2024). Demographic and socioeconomic variables that were controlled for included age (18–34 years, 35–54 years, or 55 years and over), gender (men or women), immigrant status (immigrant or non-immigrant), and total household income (quintiles plus missing category). Immigration status and income were included as they are well-established social determinants of health, whereby immigrants and low-income families face increased health disparities and limited access to health care (Castañeda et al., 2015; Marmot, 2005).

### Statistical Analysis

Summary statistics describing the characteristics and work environments of health care workers of LTCS homes in Canada were calculated. Chi-squared tests were conducted to compare the differences in characteristics between nurses and PSWs. To assess the associations between job strain working conditions and self-reported changes in health, multivariable logistic regression models were employed. Given variations in workload intensity and job control across occupations, analyses were conducted separately for nurses (*n* = 571) and PSWs (*n* = 1,391). Separate models were examined for each health outcome and stratified by occupation (nurses and PSWs). Prior to model estimation, multicollinearity was assessed for the three job strain indicators (working overtime, increased workloads, beyond-role work) using the Variance Inflation Factor (VIF). The VIF for all variables was less than 5, indicating no multicollinearity concerns (Gujarati, 2004). Theoretically based regression modeling was conducted to monitor changes in each model’s goodness-of-fit to the outcomes of changes in general and mental health (Kleinbaum et al., 2013). Independent variables were divided into five sets. Final models were adjusted for (1) three job strain-related working conditions, (2) demographics: age, gender, immigrant status, (3) socioeconomic factors: total household income, (4) work experience factors: number of years worked and number of locations worked, (5) work environment factors: facility ownership status and exposure to precarious work. Akaike’s Information Criterion (AIC) was calculated for each model to enable goodness-of-fit comparisons and infer the relevance of added parameters. All analyses were conducted in SAS (version 9.4) and included sample weights and bootstrap weights to account for the complex survey design and for variance estimation. Statistical significance was assessed at the 95% confidence level (*p* < .05).

## Results

### Descriptive Characteristics

The study sample (*N* = 1,962) of health care workers included nurses (29%) and PSWs (71%) employed in LTCS homes (Table 1). Most nurses (93%) and PSWs (90%) were women, and approximately half were between the ages of 35 and 54 years old (*p* < .0001). Over 37% of PSWs were over the age of 55, compared to only 23% of nurses (*p* < .0001). A higher proportion of PSWs were immigrants, compared to nurses (45% vs 42%, respectively; *p* = .0319). Total household income levels were lower on average for PSWs compared to nurses, with 44% of PSWs earning less than $70,000 compared to 21% of nurses (*p* < .0001). Most of the sample worked in a single residential care facility during the pandemic, with 18% of nurses and 11% of PSWs reporting they worked in two or more LTCS homes (*p* < .0001). Most nurses and PSWs worked in private facilities (46%), whereas more PSWs than nurses reported they did not know the facility’s ownership status (20% vs 16%, respectively; *p* = .0005). Over half of the respondents resided in Ontario and Quebec (*p* < .0001). Overall, a higher proportion of nurses than PSWs reported worsened general health (42% vs 27%, respectively) and worsened mental health (45% vs 33%, respectively) since the start of the COVID-19 pandemic (*p* < .0001). Only 11% of nurses and 12% to 15% of PSWs reported improved general and mental health (*p* < .0001). The remaining health care workers reported no changes in mental health and general health, respectively.

**Table 1.** Characteristics and Health Outcomes of Health Care Workers in Canada’s Long-Term Care and Senior Homes During the Pandemic, Nurses and Personal Support Workers (2021)

Characteristic	Nurses		Personal support workers		*p*-Value
Sample size	571		1,391		
Weighted *N*^a^	69,700		112,400		
Characteristic	*n* ^a^	%^a^	*n* ^a^	%^a^	
Gender					
Women	64,700	92.9	101,400	90.3	<.0001
Men	5,000	7.1	10,900	9.7	
Age group (years)					
18–34	16,700	24.0	14,700	13.1	<.0001
35–54	37,300	53.5	55,900	49.8	
55 and over	15,700	22.6	41,800	37.2	
Immigration status					
Non-immigrant	39,600	57.6	59,300	54.7	.0319
Immigrant	29,200	42.4	49,100	45.3	
Total household income					
Below $40,000	5,300	7.7	15,500	13.8	<.0001
$40–69,000	9,100	13.1	33,400	29.7	
$70–99,000	16,600	23.8	23,500	20.9	
$100–149,000	16,500	23.7	16,300	14.5	
$150,000 and over	11,400	16.4	5,700	5.0	
Not reported	10,700	15.4	18,100	16.1	
Number of years working					
0–4 years	15,100	22.1	14,900	13.7	<.0001
5–14 years	29,500	43.1	50,400	46.3	
15 or more years	23,900	34.8	43,500	40.0	
Number of locations worked					
Only 1 location	57,100	82.0	100,400	89.5	<.0001
2 or more locations	12,600	18.0	11,800	10.6	
Ownership of facility					
Public	25,500	38.0	37,500	34.3	.0005
Private	31,000	46.1	50,200	46.0	
Do not know	10,600	15.9	21,400	19.6	
	Nurses		Personal support workers		*p*-Value
Sample size	571		1,391		
Weighted *N*^a^	69,700		112,400		
Characteristic	*n* ^a^	%^a^	*n* ^a^	%^a^	
Province					
Atlantic Provinces (NB, NL, NS, PEI)	5,800	8.3	8,300	7.4	<.0001
Quebec	17,300	24.9	25,200	22.5	
Ontario	28,500	41.0	43,800	39.0	
Manitoba	3,100	4.5	4,900	4.3	
Saskatchewan	1,700	2.5	4,500	4.0	
Alberta	5,900	8.4	11,000	9.8	
British Columbia	7,200	10.4	14,600	13.0	
Outcome	*n* ^a^	%^a^	*n* ^a^	%^a^	*p*-Value
Changes in general health					
Better now	7,400	10.8	13,400	12.2	<.0001
No change	32,900	47.7	66,800	60.8	
Worse now	28,600	41.5	29,700	27.0	
Changes in mental health					
Better now	7,300	10.7	16,700	15.2	<.0001
No change	30,500	44.3	56,600	51.6	
Worse now	30,900	45.0	36,400	33.2	

*Source.* Survey on Health Care Workers’ Experiences During the Pandemic (SHCWEP), 2021.

*Note.* NB = New Brunswick; NL = Newfoundland and Labrador; NS = Nova Scotia; PEI = Prince Edward Island.

a In accordance to the SHCWEP reporting guidelines, weighted estimates are rounded to the nearest 100.

### Prevalence of Job Strain

**Figure 1.**
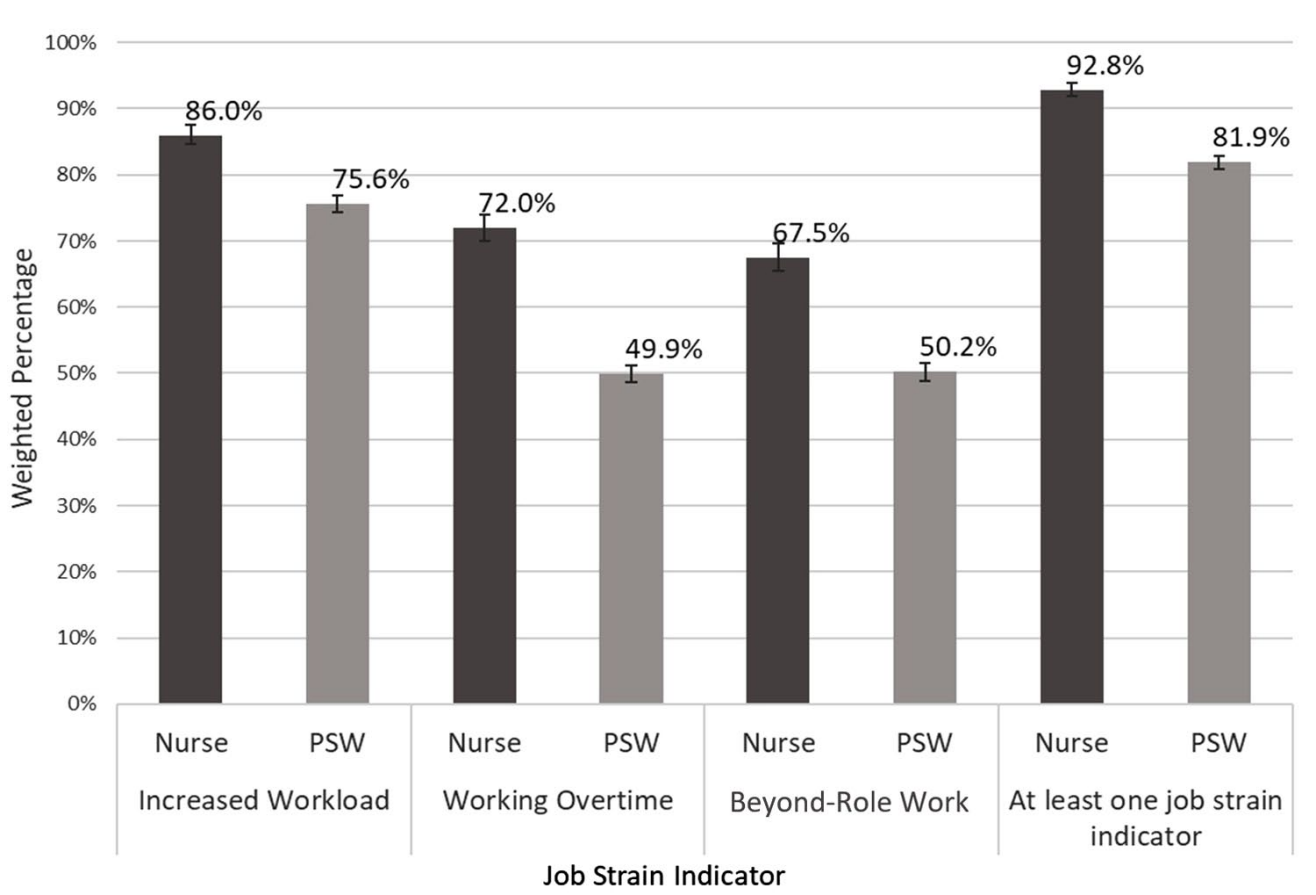
Prevalence of nurses and personal support workers (*N* = 1,962) reporting job strain indicators: increased workloads, overtime work, and beyond-role work in Canada’s LTCS home sector, 2021

The prevalence of job strain during the pandemic was high, with 93% (95% Confidence Interval [CI] 91.8%, 93.8%) of nurses and 82% (95% CI [80.9%, 82.9%]) PSWs reporting at least one job strain-related working condition including increased workloads, more hours of work, or beyond-role work (Figure 1). Consistently, more nurses reported experiencing these strenuous working conditions compared to PSWs. More than two-thirds of nurses (68%; 95% CI [65.44%, 69.6%]) and half of PSWs (50%; 95% CI [48.8%, 51.5%]) reported working beyond their usual role and responsibilities (beyond-role work). Approximately, 72% (95% CI [70.0%, 74.0%]) of nurses and 50% (95% CI [48.6%, 51.2%]) of PSWs indicated working more hours than usual. Most notably, 86% (95% CI [84.6%, 87.5%]) of nurses and 76% (95% CI [74.4%, 76.8%]) of PSWs reported they had increased workloads during the pandemic.

### Bivariate Regression Analysis

To determine associations between the job strain-related working conditions and changes in health outcomes for nurses and PSWs, bivariate logistic regression analyses were conducted to examine crude unadjusted odds ratios, overall and by occupation (Table 2). Overall, each job strain-related working condition was positively associated with worsened general and mental health for health care workers in LTCS homes. For example, bivariate regressions revealed among nurses, those who reported increased workloads had three times higher odds of experiencing worsened mental health compared to nurses who did not report increases in their workload during the pandemic. Similarly, PSWs with increased work had 85% increased odds of reporting worsened mental health compared to their counterparts without workload increases. By occupation, these positive associations remained, with nurses exhibiting a higher unadjusted odds ratio compared to PSWs for each relationship.

**Table 2.** Crude Unadjusted Odds Ratios Examining the Association Between Job Strain Exposures and Changes in Health Outcomes, by Occupation, 2021

Health outcome	All	Nurses	PSWs
	*OR* [95% CI]	*OR* [95% CI]	*OR* [95% CI]
*Worsened general health*			
Increased workload			
Yes	3.37 [2.90, 3.91]	5.93 [4.22, 8.34]	2.37 [2.00, 2.80]
No (ref)	1.00	1.00	1.00
Working overtime			
Yes	1.95 [1.75, 2.17]	1.92 [1.56, 2.37]	1.65 [1.46, 1.86]
No (ref)	1.00	1.00	1.00
Beyond-role work			
Yes	1.63 [1.46, 1.82]	1.74 [1.40, 2.14]	1.34 [1.18, 1.51]
No (ref)	1.00	1.00	1.00
*Worsened mental health*			
Increased workload			
Yes	2.28 [1.98, 2.63]	3.03 [2.27, 4.06]	1.85 [1.57, 2.17]
No (ref)	1.00	1.00	1.00
Working overtime			
Yes	1.73 [1.56, 1.92]	1.80 [1.48, 2.20]	1.49 [1.33, 1.67]
No (ref)	1.00	1.00	1.00
Beyond-role work			
Yes	1.97 [1.78, 2.18]	2.09 [1.70, 2.56]	1.72 [1.54, 1.93]
No (ref)	1.00	1.00	1.00

*Source.* Survey on Health Care Workers’ Experiences During the Pandemic (SHCWEP), 2021.

*Note.* CI = confidence interval; *OR* = odds ratio.

### Multivariable Regression Analysis

Multivariable logistic regressions were conducted to examine the relationships between job strain working conditions and changes in health for nurses and PSWs, controlling for the presence of job-strain covariates, as well as sociodemographic and employment characteristics in the final model through theoretically-based regression modeling approaches (Table 3).The AIC decreased upon the addition of each set of variables in the models, with the final model demonstrating the best overall fit, having the lowest AIC. The two measures of workload intensity—increased workloads and overtime work—were both positively associated with worsened general health for nurses and PSWs during the pandemic. Nurses who experienced increased workloads had nearly six times higher odds of reporting worsening general health compared to nurses reporting no workload increases. Among PSWs, there was a smaller but statistically significant positive association between workload increases and worsened general health (two times higher odds). Nurses and PSWs had a 29% and 33% increased odds of worsened general health, respectively, when working overtime compared to those who did not work more hours.

**Table 3.** Adjusted Odds Ratios for Worsened Self-Reported General Health and Mental Health Among Health Care Workers in Canada’s LTCS Homes, by Occupation, 2021

Outcome	Worsened general health		Worsened mental health	
Variable	Nurses	PSWs	Nurses	PSWs
	*OR* [95% CI]	*OR* [95% CI]	*OR* [95% CI]	*OR* [95% CI]
Increased workload				
Yes	5.73* [4.10, 8.01]	2.10* [1.72, 2.56]	2.13* [1.52, 2.98]	1.57* [1.29, 1.91]
No (Ref)	1.00	1.00	1.00	1.00
Working overtime				
Yes	1.29* [1.02, 1.62]	1.33* [1.16, 1.51]	1.16* [0.92, 1.47]	1.03 [0.91, 1.17]
No (Ref)	1.00	1.00	1.00	1.00
Beyond-role work				
Yes	1.25* [1.01, 1.56]	1.04 [0.90, 1.19]	1.76* [1.42, 2.18]	1.46* [1.27, 1.68]
No (Ref)	1.00	1.00	1.00	1.00
Gender				
Men (Ref)	1.00	1.00	1.00	1.00
Women	0.67* [0.49, 0.92]	1.30* [1.03, 1.64]	1.05 [0.81, 1.35]	1.10 [0.89, 1.37]
Age (years old)				
18–34 (Ref)	1.00	1.00	1.00	1.00
35–54	0.98 [0.74, 1.31]	1.38* [1.12, 1.69]	0.57* [0.44, 0.73]	0.58* [0.47, 0.71]
5 and over	1.02 [0.73, 1.43]	1.13 [0.88, 1.43]	0.57* [0.41, 0.79]	0.38* [0.30, 0.49]
Immigrant status				
Non-immigrant (Ref)	1.00			
Immigrant	1.01 [0.82, 1.24]	0.80* [0.69, 0.92]	0.89 [0.73, 1.09]	0.41* [0.36, 0.47]
Total household income				
$150,000 and over (Ref)	1.00	1.00	1.00	1.00
$100–149,000	0.38* [0.29, 0.50]	0.84 [0.58, 1.22]	0.74* [0.56, 0.97]	1.21 [0.85, 1.71]
$70–99,000	0.30* [0.22, 0.41]	1.59* [1.12, 2.26]	0.70* [0.52, 0.93]	1.16 [0.83, 1.63]
$40–69,000	0.39* [0.29, 0.52]	1.08 [0.76, 1.54]	0.98 [0.71, 1.33]	1.28 [0.92, 1.77]
Below $40,000	0.08* [0.05, 0.15]	1.03 [0.71, 1.50]	0.50* [0.33, 0.75]	1.18 [0.83, 1.67]
Not reported	0.32* [0.21, 0.48]	1.11 [0.77, 1.60]	0.47* [0.30, 0.74]	1.39 [0.98, 1.98]
Number of years worked				
0–4 years (Ref)	1.00	1.00	1.00	1.00
5–14 years	1.24 [0.90, 1.69]	1.51* [1.14, 1.99]	1.16 [0.89, 1.51]	1.07 [0.84, 1.36]
15 years and over	1.95* [1.47, 2.57]	1.50* [1.18, 1.93]	0.72* [0.53, 0.98]	1.44* [1.17, 1.77]
Number of locations worked				
One location (Ref)	1.00	1.00	1.00	1.00
2 or more	0.71* [0.56, 0.90]	0.99 [0.79, 1.23]	0.86 [0.69, 1.08]	1.13 [0.92, 1.40]
Facility ownership status				
Public (Ref)	1.00	1.00	1.00	1.00
Do not know	1.41* [1.00, 1.97]	1.32* [1.10, 1.57]	0.70* [0.51, 0.95]	0.91 [0.77, 1.07]
Private	1.01 [0.84, 1.23]	0.68* [0.59, 0.79]	0.74* [0.61, 0.90]	0.68* [0.59, 0.79]
Work precarity				
Yes	2.78* [1.61, 4.81]	2.63* [2.07, 3.34]	0.72 [0.42, 1.21]	1.63* [1.21, 2.21]
No (Ref)	1.00	1.00	1.00	1.00

*Source.* Survey on Health Care Workers’ Experiences During the Pandemic (SHCWEP), 2021.

Note. CI = confidence interval; OR = odds ratio; PSWs = Personal Support Workers.

* Statistically significantly different from the reference group (p < .05).

There were differences in the relationship between beyond-role work and worsened general health between nurses and PSWs. Nurses who worked outside their usual responsibilities exhibited 25% increased odds of reporting worsened general health, compared to those that did not work beyond their role. Beyond-role work, however, was not statistically significantly associated with worsened general health for PSWs (Table 3).

For mental health, beyond-role work was significantly associated with worsened outcomes for both groups. Nurses and PSWs who were required to work outside of their typical responsibilities had 76% and 46% increased odds, respectively, of reporting deteriorated mental health since the onset of the pandemic, compared to their counterparts who did not engage in beyond-role work. Working overtime was not associated with worsened mental health for nurses or PSWs during the pandemic, after controlling for all covariates. However, exposures to increased workloads remained statistically significantly associated with worsened mental health for nurses and PSWs, presenting an adjusted odds ratio of 2.13 and 1.57, respectively (Table 3).

Finally, some covariates showed notable associations. After controlling for job strain and other characteristics, nurses and PSWs exposed to precarious work had over 2.5 times the odds of reporting worsened general health than LTCS workers not exposed to precarity. Furthermore, PSWs working in precarious conditions had 63% increased odds of experiencing worsened mental health compared to those exposed to more stable working conditions, although this association was not statistically significant for nurses (Table 3).

### Sensitivity Analysis

Two sensitivity analyses were conducted. The first analysis examined whether associations between job strain and health changes differed if workers who reported improved health were excluded from the sample. The analysis revealed that little to no difference in results with or without the inclusion of the group. Therefore, these respondents were included in the study cohort to preserve the sample and maintain outcome and covariate distributions. A second sensitivity analysis investigated whether the association between job strain and changes in health during the pandemic could be explained by current mental or physical health status at the time of survey. The analysis revealed the relationship between job strain and changes in health remained unchanged. As a result, current health status was not included in the final models.

## Discussion

This study examined the prevalence of job strain-related working conditions and their association with health changes, exploring differences between nurses and PSWs. The findings demonstrate job strain-related working conditions were experienced by a high proportion of health workers in LTCS facilities during the pandemic, with over 90% of nurses and 80% of PSWs reporting at least one indicator, in the form of increased workloads, overtime work, or beyond-role work. Consistently, more nurses reported experiencing these conditions compared to PSWs. Furthermore, a higher proportion of nurses than PSWs reported deteriorating general health and mental health relative to before the pandemic.

Interestingly, when comparing to the literature, only one study conducted among rural LTC homes in Saskatchewan, Canada described contrasting findings, reporting PSWs had greater job strain than nurses (Morgan et al., 2002). These findings are limited in generalizability and used data collected over 20 years ago, which do not account for the evolution of work experiences in LTCS homes among staff over time, particularly during a pandemic. The current research study provides a more generalizable and updated analysis on job strain experienced by regulated and unregulated workers in LTCS settings. To our knowledge, this may be the first study to report on job strain working conditions and workers’ health changes across Canada’s LTCS sector during the pandemic.

Existing research reveals that the pandemic exacerbated pre-existing poor working conditions in LTC homes, creating heightened staffing challenges and increased workloads due to disproportionately high resident morbidity and mortality, and increased absenteeism following illness or fear of contracting and spreading the virus (Boamah et al., 2023; CIHI, 2021; Comas-Herrera et al., 2020; Fisman et al., 2020; Thompson et al., 2020). These inadvertent consequences of increased workloads may include working overtime to meet residents’ needs (Yau et al., 2021). This study adds to the literature by revealing the increased workloads were associated with worsened general and mental health among direct care workers. Furthermore, working overtime was associated with deteriorating general health for nurses and PSWs working in LTCS homes, but not mental health. As such, other job strain working conditions, such as increased workloads and engaging in beyond-role work, may better predict worsened mental health for LTCS workers.

Notably, this study found the extent of beyond-role work experienced during the pandemic was greater among nurses than PSWs, and was associated with worsened general health among nurses, but not PSWs. In Canada’s residential care sector, registered nurses and nurse practitioners often hold managerial positions, supervising the provision of clinical care and administrative tasks by registered practical nurses, or more physically demanding personal care by PSWs (Braedley et al., 2018). During the pandemic, severe understaffing led regulated workers, such as nurses, deviate from the usual hierarchical organization of care and engage in more team-based approaches, where these workers participated in work outside of their traditional responsibilities to meet the high demands for resident care (Hung et al., 2022; McGilton et al., 2021). Moreover, reduced levels of job control may have been experienced by nurses when faced with rapidly evolving pandemic guidelines (Nizzer et al., 2025). Several studies suggest shifting guidance resulted in workloads outside of expected roles for nurses that involved more administrative tasks such as managing visitors and distancing protocols, supporting resident-family communication, and training new staff from temp agencies (Boamah et al., 2023; Lightman, 2022; McGilton et al., 2021; Reynolds et al., 2022). These unplanned shifts in roles, additional tasks, and overtime work may have led to exhaustion, injury, and ultimately, worsening physical and mental health for nurses during the pandemic. Ongoing losses of LTC nursing staff demonstrates the immediate need to address these strenuous working conditions to improve health and support retention (CIHI, 2024).

PSWs also engaged in tasks outside of their responsibilities and these experiences of low job control were associated with worsened mental health. However, beyond-role work was not associated with worsened general health, which may reflect the historical nature of role expansion in this unregulated workforce. PSWs often adapted to broad and inconsistent responsibilities even before the pandemic (Marani et al., 2021; Saari et al., 2018). Variation in PSW roles have been attributed to the lack of skill-based expertise in the occupation or governing body overseeing standards for PSW knowledge, skills, and scope of services provided (Marani et al., 2021). As personal support work is not regulated, there is inconsistency in training requirements by employers across the country, with no national guidance on training expectations, competency profiles, job titles, ethics of practice or disciplinary procedures (CIHI, 2023; Marani et al., 2021). These conditions often exposed PSWs to precarious working conditions, including casual or part-time employment, low wages, and limited benefits (Marani et al., 2021; Zagrodney & Saks, 2017). Differences in training and experience may also increase the supervisory work carried out by nurses, especially during the pandemic when adequate infection, prevention, and control training was essential.

Considering a small proportion of nurses work in a single facility and the mounting complexity of resident care needs over time (Ng et al., 2020), increasing nursing staffing levels depending on the number of residents per home and reorganizing hours of work to ensure decent working conditions, could reduce job strain among LTCS workers. Higher registered nurse staffing levels are associated with improved resident quality of care in US nursing homes (Castle & Engberg, 2007). With an adequate nurse-to-resident ratio, the distribution of decision-making authority, and medically oriented care provision can be shared among more than just a few health professionals. Implementing adequate nurse/resident ratios and reorganizing staff working hours to reduce workloads, exposure to unplanned and ambiguous work, and overtime work could improve workers mental and general health.

Furthermore, while six provinces created job training and education standards for PSWs (CIHI, 2023), expanding this guidance nationally and mandating the implementation of these standards consistently across jurisdictions may ease the job strain experienced by LTCS workers. Sustained investment and innovations in training and upskilling as well as shifts in workplace culture are needed to increase PSW job autonomy and empower them to participate in decisions about providing resident care (Purcell et al., 2024). Finally, workers safety can be prioritized in the sector by ensuring the availability of organizational resources and supports such as consistent PPE supplies and regulatory advances to monitor facility staffing levels (Kung et al., 2024; Purcell et al., 2024). Staff shortages have compromised the safety and wellbeing of LTCS workers, in addition to impeding the provision of quality resident care (Berta et al., 2022). As such, the implementation of formal mechanisms to monitor staffing levels by staff skill mix across LTCS homes may improve working conditions over time. These approaches may reduce workloads and overtime experienced by LTCS home employees, who have been and continue to remain short-staffed and under-resourced (CIHI, 2024; Estabrooks et al., 2020).

### Strengths and Limitations

The strengths of this study include its nationally representative, cross-provincial sample of LTCS workers and its comparison of nurses and PSWs. It is one of the first Canada-wide studies to examine the prevalence of job strain-related working conditions among the LTCS workforce during the pandemic and their association with worsened general and mental health. Furthermore, this study reveals the work and health experiences of PSWs, unregulated workers who are often understudied due to the lack of provincial registries facilitating data collection of this essential workforce (CIHI, 2023).

There are also limitations of this study. There may be non-response bias and recall bias due to the moderate response rate and self-reported measures of health changes and job strain. While job strain has been measured using questionnaires developed by Karasek (1979) and others (Karasek et al., 1998), the SHCWEP (Statistics Canada, 2022) did not capture all related items; only 3 of 14 items were examined, including high workloads, working overtime, and beyond-role work. Omitted variable bias may exist, as facility-level characteristics such as staffing levels were not collected.

Differences between LTC and seniors’ homes that could not be examined because the survey combined workers from both settings. LTC homes are owned by a mix of public and private entities, catering to residents who require high levels of nursing care. In contrast, senior homes are owned mostly by private organizations, housing residents that require fewer supports and lower levels of care provision. Given these differences, research distinguishing between workers employed in different residential care settings should be conducted in the future. There are also limitations in the ability to infer causality. Finally, as the survey was conducted during the pandemic, findings may be generalized to nurses and PSWs working under COVID-19 or similar outbreak conditions. Internationally, these results may offer contextual insights for residential care sectors with comparable organization and mixes of care provision by regulated and unregulated health workers.

### Implications for Occupational Health Practice

Overall, more nurses than PSWs experienced worsened health and job strain, characterized by increased workloads, overtime work, and beyond-role work during the COVID-19 pandemic. Worsened general and mental health was associated with workload increases for direct care workers in the LTCS sector. Beyond-role work and overtime work were associated with worsened mental health for nurses and PSWs. However, beyond-role work was associated with deteriorating general health for nurses but not for PSWs. Differing roles, expectations, regulations, and experiences during and before the pandemic may have contributed to job strain and health outcome differences across workers in different health occupations. These findings offer important insights on how strenuous working conditions are associated with different health outcomes for workers in unique ways, providing opportunities for enhancing the sector’s workplace safety measures in the future. Occupational health practitioners are uniquely positioned to identify when these job-related working conditions are excessive or unbalanced. Furthermore, targeted monitoring of mental and general health among nurses and PSWs in LTCS settings is needed for early detection by practitioners to prevent job strain and subsequent turnover effects. By understanding role-specific stressors, occupational health practitioners can work with management to review and redesign jobs with manageable workloads and limited overtime to ensure the health and safety of the LTCS workforce.

Considering the ongoing shortage of health care workers in the LTCS sector (CIHI, 2024), it is imperative to understand how experiences of job strain during the pandemic may have influenced health care workers’ intention to leave the sector and affected their economic, social, and health outcomes in the long-term. As such, further research is needed to explore the experiences of nurses and PSWs with high workloads and low job control. While this study offers key insights into job strain and health outcomes of regulated and unregulated workers, more research is needed to examine how changes to the organization of care, facility-level regulations, and distribution of labor impact the health of LTCS workers by occupation.

**Applications to Professional Practice**

This research study suggests that there are differences in job strain-related working conditions experienced by regulated (e.g., nurses) and unregulated (e.g., PSWs) health care workers in LTCS settings. More nurses than PSWs reported strenuous working conditions in Canada, with high workloads, more hours and working beyond their expected role during the pandemic. Increased workloads for both nurses and PSWs were related to worsened general and mental health. However, beyond-role work was associated with worsened general health for nurses but not PSWs. Implementing adequate nurse/resident ratios could improve regulated workers’ health by distributing the decision-making authority to more nursing professionals, thereby reducing workloads, beyond-role circumstances, overtime work, and turnover, however further research is needed. By understanding role-specific stressors, occupational health practitioners are uniquely positioned to identify and monitor experiences of job strain among direct care staff as well as providing counsel for solutions by collaborating with management to ensure the health and safety of the LTCS workforce. Considering the ongoing health care worker shortage, future research can build on the results of this study to examine how changes to the organization of care can impact workers’ health.
